# COVID-19 and long-term impact on symptoms and Health-Related Quality of Life in Costa Rica: the RESPIRA cohort study

**DOI:** 10.1186/s12879-024-09450-6

**Published:** 2024-06-04

**Authors:** Cristina Barboza-Solis, Romain Fantin, Allan Hildesheim, Ruth Pfeiffer, Carolina Porras, Julia Butt, Tim Waterboer, Henriette Raventós, Arturo Abdelnour, Amada Aparicio, Viviana Loria, D. Rebecca Prevots, Mitchell H. Gail, Rolando Herrero, the RESPIRA Study Group, Alejandro Calderón, Karla Moreno, Melvin Morera, Roy Wong, Roberto Castro, Bernal Cortés, Rebecca Ocampo, Michael Zúñiga, Juan Carlos Vanegas, Kaiyuan Sun, Marco Binder

**Affiliations:** 1 Facultad de Odontología, Universidad de Costa Rica, San José, Costa Rica; 2 Agencia Costarricense de Investigaciones Biomédicas, Fundación INCIENSA, San José, Costa Rica; 3 Biostatistics Branch, Division of Epidemiology and Genetics, National Cancer Institute, Bethesda, MD USA; 4 Epidemiology and Population Studies Unit, Division of Intramural Research, National Institute of Allergy and Infectious Diseases, Rockville, MD USA; 5 Division of Infections and Cancer Epidemiology, German Cancer Research Center (DKFZ), Heidelberg, Germany; 6 Caja Costarricense de Seguro Social, San José, Costa Rica; 7 Centro de Investigación en Biología Celular y Molecular, Universidad de Costa Rica, San José, Costa Rica; 8 Escuela de Biología, Universidad de Costa Rica, San José, Costa Rica; 9 Division of Cancer Epidemiology and Genetics, National Cancer Institute, 9609 Medical Center Drive RM 7-E138, MSC 9780, Bethesda, MD 20892 USA

**Keywords:** Post-Acute Sequelae of SARS-CoV-2, PASC, Post-COVID Conditions, PCC, Middle-Income Country, Cohort study, Health-Related Quality of Life, HRQoL, symptoms, Costa Rica

## Abstract

**Background:**

Evidence continues to accumulate regarding the potential long-term health consequences of COVID-19 in the population. To distinguish between COVID-19-related symptoms and health limitations from those caused by other conditions, it is essential to compare cases with community controls using prospective data ensuring case-control status. The RESPIRA study addresses this need by investigating the lasting impact of COVID-19 on Health-related Quality of Life (HRQoL) and symptomatology in a population-based cohort in Costa Rica, thereby providing a robust framework for controlling HRQoL and symptoms.

**Methods:**

The study comprised 641 PCR-confirmed, unvaccinated cases of COVID-19 and 947 matched population-based controls. Infection was confirmed using antibody tests on enrollment serum samples and symptoms were monitored monthly for 6 months post-enrolment. Administered at the 6-month visit (occurring between 6- and 2-months post-diagnosis for cases and 6 months after enrollment for controls), HRQoL and Self-Perceived Health Change were assessed using the SF-36, while brain fog, using three items from the Mental Health Inventory (MHI). Regression models were utilized to analyze SF-36, MHI scores, and Self-Perceived Health Change, adjusted for case/control status, severity (mild case, moderate case, hospitalized) and additional independent variables. Sensitivity analyses confirmed the robustness of the findings.

**Results:**

Cases showed significantly higher prevalences of joint pain, chest tightness, and skin manifestations, that stabilized at higher frequencies from the fourth month post-diagnosis onwards (2.0%, 1.2%, and 0.8% respectively) compared to controls (0.9%, 0.4%, 0.2% respectively). Cases also exhibited significantly lower HRQoL than controls across all dimensions in the fully adjusted model, with a 12.4 percentage-point difference [95%CI: 9.4-14.6], in self-reported health compared to one year prior. Cases reported 8.0% [95%CI: 4.2, 11.5] more physical limitations, 7.3% [95%CI: 3.5, 10.5] increased lack of vitality, and 6.0% [95%CI: 2.4, 9.0] more brain fog compared to controls with similar characteristics. Undiagnosed cases detected with antibody tests among controls had HRQoL comparable to antibody negative controls. Differences were more pronounced in individuals with moderate or severe disease and among women.

**Conclusions:**

PCR-confirmed unvaccinated cases experienced prolonged HRQoL reductions 6 months to 2 years after diagnosis, this was particularly the case in severe cases and among women. Mildly symptomatic cases showed no significant long-term sequelae.

**Supplementary Information:**

The online version contains supplementary material available at 10.1186/s12879-024-09450-6.

## Key message

1. Study design, methodology for symptoms attribution: The study introduces employs a methodology that enables the attribution of symptoms and enduring health impacts specifically to COVID-19. This method allows for the disentanglement of COVID-19-related symptoms and health limitations, from those caused by other conditions, providing a clearer understanding of the disease's symptomatology.
2. Enduring Physical and Mental Health Impacts: The study reveals that COVID-19 has enduring physical and mental health impacts on non-vaccinated individuals, persisting from 6 months to 2 years post-diagnosis. These effects include a lasting reduction in health-related quality of life, characterized by greater physical limitations, reduced vitality, and increased cognitive impairment ("brain fog").
3. Differential impact based on gender and severity: Women and individuals with severe cases of COVID-19 were found to be the most affected, experiencing significant and lasting health consequences. In contrast, those with mild or undiagnosed cases did not suffer long-term health effects.

## Introduction

Evidence continues to accumulate on the potential long-term health consequences of COVID-19 [1–3]. Since a large proportion of the world population has now been infected, in an effort to classify these consequences, the terms Post-COVID Conditions (PCC) [4, 5] or Post-Acute Sequelae of SARS-CoV-2 (PASC) were introduced, defined as a group of new, ongoing or relapsing symptoms associated with a COVID-19 infection, present 30 or more days after infection [5, 6]. The WHO has characterized that PCC “occurs in individuals with a history of probable or confirmed SARS-CoV-2 infection, usually 3 months from the onset of COVID-19 with symptoms that last for at least 2 months and cannot be explained by an alternative diagnosis” [7]. The main symptoms of PCC include fatigue, shortness of breath, altered taste or smell, muscle/joint pain, brain fog, chest pain, cough, and headache [8–10]. PCC is more prevalent in females [11], individuals with higher BMI, those unvaccinated [12], and those who had severe COVID-19 [13]. The earliest studies were based on hospitalized survivors [14], but recent reports indicate that moderate and mild cases might also have a delayed health impact.

PCC prevalence in recovered COVID-19 cases varies considerably across studies [1, 15–17], depending on definition, time since infection, and disease severity. These discrepancies have hampered estimation of the actual long-term impact of COVID-19 on a population’s general health. Furthermore, it remains unclear whether self-reported symptoms after initial recovery from infection can be directly attributed to COVID-19 or result from other, concomitant conditions. Recently, a six-month study of 10,000 individuals with COVID-19 found that 10% of participants in the US were classified as PASC positive using a score [6]. However, there is limited evaluation of long-term sequelae of mild/moderate COVID-19 in a population-based group of patients [18], compared to matched community controls, especially in Low- and Middle-Income Countries (LMICs).

Health Related Quality of Life (HRQoL) assesses overall health via the SF-36 questionnaire, considering physical limitations, mental health, emotional and social well-being [19]. It captures how reduced physical and mental health impacts daily activities. Studies have shown that HRQoL, as measured by SF-36 [13], can predict subsequent morbidity and mortality [20–22].

We assessed the enduring effects of COVID-19 by comparing the HRQoL and symptoms of individuals diagnosed with COVID-19, 6 to 25.8 months post-infection, with a control group from the RESPIRA cohort study in Costa Rica. Cases and controls were matched on age, sex, residence, and recruitment time. Adjusting for common confounding allowed to distinguish differences in HRQoL and symptom prevalence between cases and community controls, shedding light on the lasting impact of COVID-19 on the population [23].

## Materials and methods

### Study population: The RESPIRA cohort [23]

Costa Rica, a middle-income Central American country of 5 million residents, operates a universal healthcare system [24] administered by the Caja Costarricense de Seguro Social (CCSS). This centralized institution offers robust vital statistics and surveillance data similar to certain high-income nations [25, 26]. Throughout the COVID-19 pandemic, the country enforced mandatory COVID-19 case reporting across public and private health facilities.

RESPIRA is a population-based prospective study of COVID-19 cases and community controls. From November 2020 to October 2021, 999 PCR-confirmed COVID-19 cases randomly selected from the national surveillance list, including all the cases diagnosed in Costa Rica in both public and private laboratories; and 1999 matched population-based controls were recruited. Cases were recruited from 48 of the 81 cantons of Costa Rica, where 70% (3.6 million persons) of the population reside. Two population-based controls matched to cases on age, sex and residence, were randomly selected for each participating case and recruited in a three-month window after identifying the case. Antibodies testing was applied in controls. Overall, 28% of cases were defined as active, recruited within two weeks of diagnosis; the remaining cases were enrolled from 15 days through 17 months after COVID-19 diagnosis by PCR-test [23, 27].

**Fig. 1:**
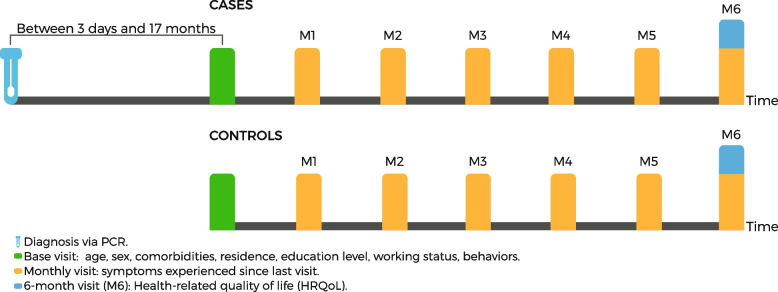
Study schematic

At recruitment, data were collected regarding socioeconomic characteristics, comorbidities (hypertension, type II Diabetes, obesity, hypercholesterolemia, asthma, and other comorbidities) [23], health behaviors, and symptoms or hospitalization during infection (to define severity in cases) (Fig. 1). During follow-up, participants were visited monthly and data were collected through an interview inquiring about a large list of symptoms (e.g. headache, cough, fatigue, fever, diarrhea, vomiting pain when swallowing, shortness of breath, muscle pain, joint pain, abdominal pain, chest tightness, nasal congestion, productive cough, and skin manifestation). At the 6-month visit, the SF-36 questionnaire (HRQoL) [28] and three questions on changes in the ability to concentrate in the Section 5-Mental Health Inventory (MHI) were administered (see study outcomes) [29]. Among cases, the 6-month visit occurred 6.0 to 25.8 months after their COVID-19 diagnosis (Median = 9.0 months).

### Exclusion criteria

In the cohort, 866 cases and 1685 controls aged 18 years or older were included in the analysis. Participants who did not complete their 6-month visit (*N*=444, 17%) or did not respond to the SF-36 questionnaire (*N*=22) were excluded. Additionally, self-declared reinfected cases (*N*=16) and controls with evidence of prior COVID-19 infection (through self-declaration at enrollment or during follow-up, identification in the national surveillance system, or positive antibodies) were excluded (*N*=448). Antibody testing (for protein N and S1-RBD) was conducted at the German Cancer Research Center (DKFZ) Heidelberg [30]. Cases vaccinated prior to infection were also excluded (*N*=38).

### Severity in cases

Cases were categorized into three groups based on the severity of their COVID-19 infection: mild, moderate, and hospitalized. Mild and moderate were determined by the presence of five symptoms associated with hospitalization in RESPIRA: fever, shortness of breath, chest tightness, disorientation/lethargy/confusion, and fatigue. Moderate cases included non-hospitalized participants with three or more of these symptoms, while those with fewer were considered mild. Asymptomatic cases were rare (*n*=15, 2.3%).

### Study outcomes

Symptoms were assessed at monthly follow-up visits, for both cases and controls, considering symptoms since the preceding visit (if it occurred within 21-60 days prior to current visit).

HRQoL (SF-36 questionnaire) was administered at the 6-month visit. The questionnaire captures health-related interference with daily life activities due to physical health limitations or emotional problems, including social activities, vitality, or symptoms associated with anxiety/depression during the past 4 weeks. The scoring algorithm was based on a factor analysis as presented by Farivar et al. [31] to convert the SF-36 raw scores into two dimensions (see Supplementary Materials), with higher scores related to better HRQoL [32]: the Physical Component Summary (PCS) and the Mental Component Summary (MCS) [33]. A concentration scale was constructed using three Mental Health Inventory (MHI) questions about memory, concentration/thinking, and reasoning/problem solving. MHI was the average score associated with response to the three questions. The PCS, MCS, and MIH were standardized (mean=50, sd=10) [31].

Self-Perceived Health Change was assessed using the SF-36 question, "*Compared to one year ago, how would you rate your health in general now?*" (not included in the PCS / MCS). Participants were categorized as either experiencing worsening health ("*Somewhat worse*" / "*Much worse*") or improving health ("*Much better*" / "*Somewhat better*" / "*About the same*"). Cases with a disease duration exceeding one year prior to the interview date were excluded when analyzing this specific outcome (*N*=209).

### Independent variables

Adjustment variables included decennial age, sex (male/female), province, education level (primary education or less / lower than secondary education / upper secondary education / higher education), working status (employee public or private / independent or informal workers / not working), current smoking status (current smoker, non-smoker), case-control status, and “Other sickness episode than COVID-19 during follow-up” was added as a control variable inquiring about symptoms that started after recruitment in the last four months in cases and in controls. It was created to investigate if cases reported systematically being sicker, on average, than controls, which could have biased the comparison. Comorbidities encompassed hypertension, diabetes, obesity, hypercholesterolemia, asthma, and those associated with a high risk of severe COVID-19 [23], alongside additional comorbidities not directly linked to COVID-19 (please see supplementary information).

### Statistical analysis

Data from controls were used to estimate the hypothetical health status cases would have had at the 6-month visit if they had not contracted COVID-19. The difference in symptom frequency and scores reflected the attributable impact of COVID-19 on health.

### Symptoms

Symptoms commonly experienced during the acute phase, such as fever, cough, and headache, typically occur within the first two weeks following diagnosis. These symptoms were assessed in both cases and controls at each monthly visit. The prevalence of symptoms was calculated based on the time since diagnosis for both cases and controls, using the percentage of visits where participants reported symptoms across all its follow-up visits.

### HRQoL items

At the 6-month visit, percentages of cases and controls reporting limitations for each SF-36 item and the MIH were calculated. Limitations were defined as scores below 25/100 for comparability across items. The proportion of cases and controls reporting at least one limitation was computed for each of the eight SF-36 dimensions (Physical Functioning, Role-Physical, Bodily Pain, General Health, Vitality, Social Functioning, Role-Emotional, Emotional Well-being). MIH was approximated using three questions, with the last two questions specifically used to describe Brain fog.

To assess the impact of COVID-19, we calculated the difference ${\Delta }_{STD}$ between the percentage of cases reporting each limitation at the 6-month visit and the estimated percentage that would have been observed in cases if they hadn't contracted the virus. To estimate ${\Delta }_{STD}$ taking into account the socioeconomic differences between cases and controls, we standardized the control group based on the cases characteristics as a reference. For each case, the following was calculated:

$${\Delta }_{STD}={p}_{CASE}-{p}_{CONTROL\_STD}$$

with

$${p}_{CASE}=P\left(Limitation=1 | Case=1\right)=1-\frac{1}{1+\text{exp}({\beta }_{0}+X\beta )}$$

$${p}_{CONTROL\_STD}=P\left(Limitation=1 | Case=0\right)=1-\frac{1}{1+\text{exp}({\beta }_{0}+{\beta }_{control}+X\beta )}$$

β were estimated using a logistic regression, in one single model combining cases and controls, adjusted for case control status and all independent variables presented above.

### HRQoL scores

The PCS, MCS, and MHI scores are continuous quantitative variables, while Self-Perceived Health Change is dichotomous. Mean scores for PCS, MCS, and MHI were computed for both controls and cases. The percentage of individuals reporting a negative Self-Perceived Health Change (*'Somewhat worse'* or *'Much worse'*) was determined for each group. These analyses were performed for the entire sample and stratified by the illness severity among cases.

### Models

Linear regression models were utilized to analyze SF-36 PCS, MCS, and MHI scores individually. The β-coefficients are presented as the slopes of the adjusted regression.

Logistic regression models were applied to assess each SF-36 item and the Self-Perceived Health Change variable, which compares current health to that of a year ago.

Two models were created for analyzing SF-36 PCS, MCS, MHI scores, and Self-Perceived Health Change. Model 1 adjusted for binary case/control status, while Model 2 included a 4-level dummy variable: control, mild case, moderate case, and hospitalized case. Models were adjusted for independent variables (see *independent variables*).

### Additional analysis

A sensitivity analysis was conducted by considering participants recruited as controls in RESPIRA who were diagnosed with COVID-19 either before recruitment (self-declared or identified in the CCSS list) (*N*=124) or after recruitment at least 30 days before the month-6 visit (*N*=153). It was expected that HRQoL of these participants would resemble that of cases. Additionally, controls without a PCR-based diagnosis but with infection detected by antibodies in enrollment serum samples were analyzed (*N*=131). The same models used in the main analysis were applied, with the exclusion of new symptom appearances.

### Ethics and data

The protocol number of this study is the R020-SABI-00261 and was approved by the Ethical Review Committee of the Center for Strategic Development and Information on Health and Social Security (CENDEISSS) of the CCSS and registered in the National Health Research Council (CONIS). All participants signed an informed consent.

## Results

**Fig. 2:**
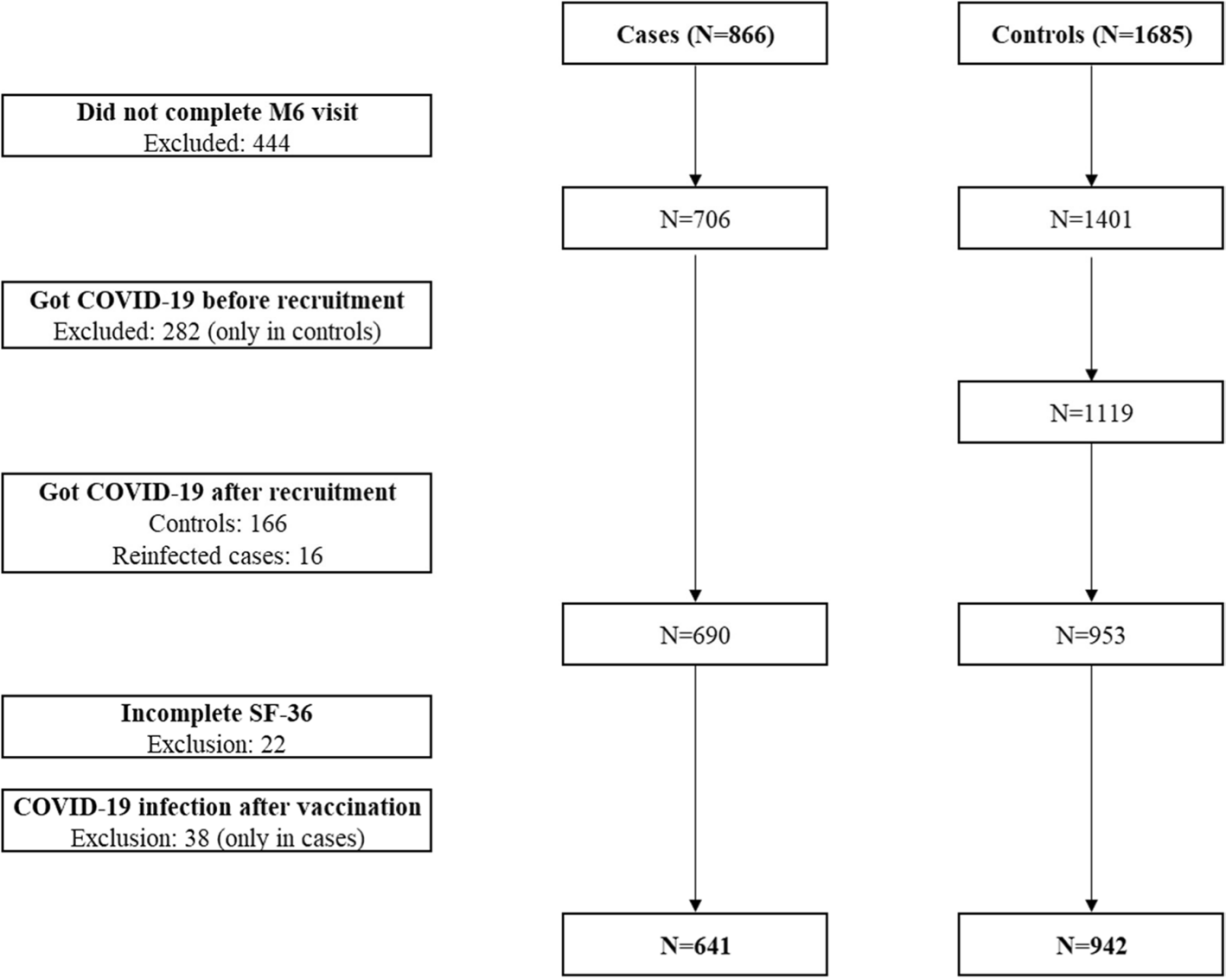
Flow-chart of the study (*N*=1583)

Figure 2 presents the study population flowchart, which began with 866 cases and 1685 controls, ultimately resulting in 641 unvaccinated cases (74% of total) and 942 controls (56% of total) for final analysis. Table 1 outlines the characteristics of the participants. Among cases, 59% were categorized as mild, 35% as moderate, and 6% as hospitalized. Controls and cases exhibited similar demographics in terms of age, sex, province of residence, and education level. A higher proportion of cases were employed compared to controls (44% vs. 20%). Although prevalence of most comorbidities was comparable between groups, asthma and obesity were slightly more prevalent among cases. Both groups experienced similar rates of other sickness episodes than COVID-19 during follow-up.

**Table 1:** Demographic and socioeconomic characteristics and comorbidities in controls and cases (NS=not significant)

	**Sample (*N*=1588)**		
	**Controls (*N*=942)**	**Cases (*N*=641)**	**Chi-2**
**Sex**			NS
Men	449 (48%)	286 (45%)	
Women	493 (52%)	355 (55%)	
**Age**			NS
18-29y	146 (16%)	117 (18%)	
30-39y	162 (17%)	121 (19%)	
40-49y	149 (16%)	119 (19%)	
50-59y	167 (18%)	116 (18%)	
60-69y	197 (21%)	106 (17%)	
70y+	121 (13%)	62 (10%)	
**Severity**			
Mild	-	379 (59%)	
Moderate	-	223 (35%)	
Hospitalized	-	39 (6%)	
**Time since diagnosis at recruitment**			
0-29 days	-	219 (34%)	
30-89 days	-	145 (23%)	
90 days or more	-	277 (43%)	
** Province**			NS
GAM	638 (68%)	419 (65%)	
Guanacaste-Puntarenas	304 (32%)	222 (35%)	
**Education level**			NS
Complete Elementary School	313 (33%)	184 (29%)	
Incomplete High School	182 (19%)	116 (18%)	
Complete High School or Technical training	197 (21%)	134 (21%)	
Complete or Incomplete University	250 (27%)	207 (32%)	
**Working status**			<0.01
Employee (public or private)	185 (20%)	283 (44%)	
Independent or informal workers	186 (20%)	98 (15%)	
Not working	571 (61%)	260 (41%)	
**Currently smoking**	122 (13%)	42 (7%)	<0.01
**Comorbidities**			
Hypertension	284 (30%)	193 (30%)	NS
Diabetes II	130 (14%)	87 (14%)	NS
Obesity	114 (12%)	101 (16%)	0.04
Cholesterol	251 (27%)	183 (29%)	NS
Asthma	106 (11%)	98 (15%)	0.02
Another comorbidity related to COVID-19	148 (16%)	85 (13%)	NS
Other comorbidity	260 (28%)	174 (27%)	NS
**Other sickness episode than COVID-19 during follow-up**^**a**^	329 (35%)	220 (34%)	NS

^a^Coming from the follow-up question

**Fig. 3:**
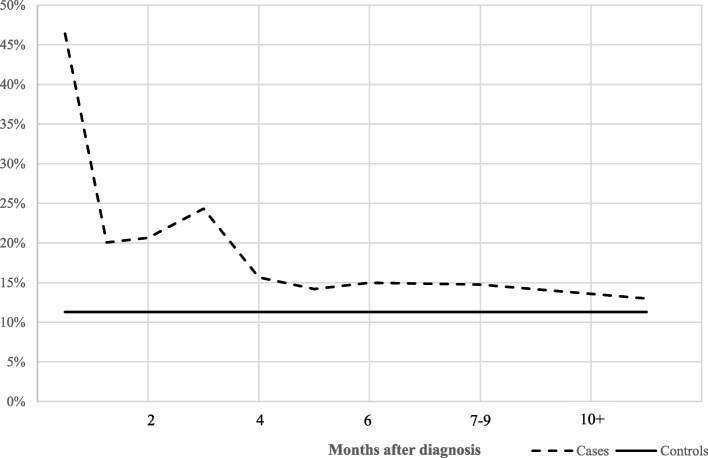
Percentage of cases and controls who declared symptoms since their last visit, according to time since diagnosis (in cases) and on average (in controls). Interpretation: On average, at two months after diagnosis, cases reported symptoms since their last visit in 20.1% of the visits. In contrast, controls reported symptoms in 11.3% of all visits during the study period

Figure 3 illustrates symptom progression associated with the acute phase in cases. 15-30 days after diagnosis, 46% reported symptoms since their last visit. This proportion decreased to 24% 1-3 months after diagnosis. Beyond the 4th month, the percentage of cases who reported symptoms since their last visit remains stable but significantly higher than in controls. On average, including all visits occurring during the 4th month and after, 14.6% of cases and 11.3% of controls reported symptoms (*p*=0.03).

Table 2 presents the COVID-19 symptoms since the last monthly visit, when this occurred at least six months post-diagnosis in cases and in controls. Symptoms reported by cases diagnosed 6 to 9 months before the visit were comparable to those diagnosed 9 months or more prior. The prevalence of common symptoms (nasal congestion, cough, headache, and pain when swallowing) was similar between cases and controls. However, cases reported joint pain (1.9% of visits), chest tightness (1.2%), skin manifestations (0.8%) are more frequently than controls (0.9%, 0.4%, and 0.2% respectively). Fatigue and shortness of breath were significantly more frequent among cases only between 9-26 months after the diagnosis.

**Table 2:** Symptoms reported by controls and cases since their last visit^a^

	**Controls**	**Cases**		
	**(*N*=934)**^**a**^	**(*N*=539)**		
**Symptom**	**All visits** **%**	**Total Cases** **%**	**Diagnosis (%)**	
			**6 to 9 months ago**	**9 to 26 months ago**
			*N***=346**	***N*=193**
At least one symptom^b^	11.3	14.0*	14.6*	13.0
Nasal congestion	5.5	5.6	5.2	6.4
Headache	4.4	4.7	4.5	5.3
Cough	3.6	4.3	4.4	4.2
Pain when swallowing	3.0	2.9	3.2	2.3
Muscle pain	2.1	2.6	2.5	2.6
Fatigue	2.0	3.0	2.7	3.6*
Productive cough	1.8	2.4	2.4	2.4
Fever	1.5	1.4	1.3	1.6
Joint pain	0.9	1.9***	2.0***	1.8*
Diarrhea	0.9	1.0	0.9	1.2
Shortness of breath	0.8	1.6	1.4	1.9*
Vomit	0.4	0.3	0.1	0.6
Abdominal pain	0.4	0.3	0.1	0.7
Chest tightness	0.4	1.2***	1.5***	0.8
Skin manifestations	0.2	0.8*	0.7	0.9*

Test between cases (total) and controls; and between cases according to time since diagnosis, were calculated (diagnosis 6-9 months vs controls and diagnosis 9-26 months vs controls)

^a^Last visit between 21 and 60 days. Mean time since last visit was 34 days in both cases and controls.

^b^The list of symptoms included the symptoms presented in the table, and other less common symptoms six months after diagnosis: loss of smell, loss of taste, red eyes, irritability, bloody vomit, palpitations, sleep disturbances

^***^p<0.01

^*^p<0.05

Table 3 displays SF-36 results by item for cases and controls, before and after standardization. Cases exhibited higher prevalence of limitations compared to standardized estimates in controls across all SF-36 and MHI items. Among those who contracted COVID-19 6 to 12 months before the 6-month visit, 20.1% reported worse health compared to a year ago (before their infection), 12.4 percentage-points higher than standardized controls. Compared to standardized controls, cases reported higher rates of physical health limitations (8% higher), fatigue (6.2% higher), feeling worn out (5.5% higher), lack of vitality (7.3% higher), feeling very nervous (4.8% higher), and brain fog (6% higher). These differences affected daily life, with 23.2% of cases reporting difficulty performing work or other activities, 8.6 percentage-points more than standardized controls.

**Table 3:** Prevalence of limitations and health problems according to the 36 items of the SF-36 and 3 items of the MHI, in cases and in controls

	**Cases** **(%)**	**Controls (%)**	**Controls**_**STD**_ **[95%CI]**	**Δ**_**STD**_ **[95%CI]**
**SF-36**				
In general, would you say your health is^a^	18.4	15.8	14.9 [11.8, 18.5]	3.6 [-0.1, 6.7]
Compared to one year ago, how would you rate your health in general now?^b,l^	20.1	8.8	7.7 [5.5, 10.7]	12.4 [9.4, 14.6]
**Does your health now limit you in these activities?**				
Vigorous activities, such as running, lifting heavy objects, participating in strenuous sports^c^	18.6	17.7	14.5 [11.6, 17.9]	4.1 [0.7, 7.0]
Moderate activities, such as moving a table, pushing a vacuum cleaner, bowling, or playing golf^c^	9.7	8.8	7.8 [5.6, 10.7]	1.9 [-1.0, 4.1]
Lifting or carrying groceries^c^	8.0	7.7	6.8 [4.8, 9.4]	1.2 [-1.4, 3.2]
Climbing several flights of stairs^c^	15.6	13.3	11.8 [9.1, 15.0]	3.8 [0.6, 6.5]
Climbing one flight of stairs^c^	7.7	7.3	6.4 [4.5, 9.0]	1.3 [-1.3, 3.3]
Bending, kneeling, or stooping^c^	13.3	11.3	10.2 [7.8, 13.1]	3.2 [0.2, 5.6]
Walking more than a mile^c^	13.0	10.0	8.6 [6.5, 11.4]	4.3 [1.6, 6.5]
Walking several blocks^c^	9.9	7.9	7.5 [5.4, 10.3]	2.4 [-0.4, 4.5]
Walking one block^c^	4.9	3.9	3.8 [2.3, 6.0]	1.2 [-1.0, 2.7]
Bathing or dressing yourself^c^	2.0	1.3	1.4 [0.6, 3.1]	1.9 [0.1, 2.7]
**During the past 4 weeks, have you had any of the following problems with your work or other regular daily activities as a result of your physical health?**				
Cut down the amount of time you spent on work or other activities^d^	19.1	16.4	14.5 [11.4, 18.2]	4.6 [0.9, 7.7]
Accomplished less than you would like^d^	27.6	23.7	22.1 [18.2, 26.5]	5.5 [1.1, 9.3]
Were limited in the kind of work or other activities^d^	22.5	19.7	18.3 [14.8, 22.4]	4.2 [0.1, 7.7]
Had difficulty performing the work or other activities (for example, it took extra effort)^d^	23.2	16.6	14.5 [11.6, 18.0]	8.6 [5.1, 11.6]
**During the past 4 weeks, have you had any of the following problems with your work or other regular daily activities as a result of any emotional problems (such as feeling depressed or anxious)?**				
Cut down the amount of time you spent on work or other activities^d^	22.2	19.3	20.2 [16.4, 24.6]	2.0 [-2.4, 5.8]
Accomplished less than you would like^d^	24.0	19.1	19.8 [16.1, 24.2]	4.1 [-0.2, 7.9]
Didn't do work or other activities as carefully as usual^d^	21.5	16.5	15.6 [12.3, 19.5]	4.9 [1.0, 8.2]
During the past 4 weeks, to what extent has your physical health or emotional problems interfered with your normal social activities with family, friends, neighbors, or groups?^e^	9.6	6.6	6.6 [4.6, 9.4]	3.0 [0.2, 5.0]
How much bodily pain have you had during the past 4 weeks?^e^	15.2	11.7	11.4 [8.6, 14.7]	3.8 [0.4, 6.5]
During the past 4 weeks, how much did pain interfere with your normal work (including both work outside the home and housework)?^e^	12.7	8.3	7.7 [5.6, 10.5]	5.0 [2.2, 7.1]
**How much of the time during the past 4 weeks**				
Did you feel full of pep?^g^	7.7	5.3	4.6 [3.0, 6.9]	3.1 [0.8, 4.7]
Have you been a very nervous person?^h^	8.8	4.4	4.0 [2.6, 6.1]	4.8 [2.7, 6.2]
Have you felt so down in the dumps that nothing could cheer you up?^h^	4.7	2.0	1.6 [0.9, 3.0]	3.1 [1.7, 3.8]
Have you felt calm and peaceful?^g^	6.4	4.4	4.0 [2.5, 6.2]	2.4 [0.2, 3.9]
Did you have a lot of energy?^g^	7.0	5.0	4.4 [2.9, 6.7]	2.7 [0.4, 4.3]
Have you felt downhearted and blue?^h^	6.0	3.6	3.5 [2.2, 5.6]	2.5 [0.4, 3.9]
Did you feel worn out?^h^	13.3	7.9	7.7 [5.6, 10.6]	5.5 [2.6, 7.7]
Have you been a happy person?^g^	4.1	4.3	3.5 [2.1, 5.8]	0.6 [-1.7, 2.0]
Did you feel tired?^h^	14.5	7.9	8.3 [6.0, 11.3]	6.2 [3.2, 8.5]
During the past 4 weeks, how much of the time has your physical health or emotional problems interfered with your social activities (like visiting with friends, relatives, etc.)?^h^	9.3	6.8	6.9 [4.8, 9.9]	2.3 [-0.7, 4.5]
**How TRUE or FALSE is each of the following statements for you.**				
I seem to get sick a little easier than other people^j^	17.0	8.6	9.6 [7.1, 12.7]	7.4 [4.3, 9.9]
I am as healthy as anybody I know^i^	17.5	12.5	11.3 [8.6, 14.6]	6.3 [3.0, 8.9]
I expect my health to get worse^j^	9.0	8.8	7.5 [5.2, 10.6]	1.5 [-1.6, 3.8]
My health is excellent^i^	14.0	10.6	9.6 [7.1, 12.8]	4.5 [1.3, 7.0]
**MHI**				
During the past month, how much of the time did you forget, for example, things that happened recently, where you put things, appointments?^h^	11.0	9.8	10.0 [7.3, 13.5]	1.2 [-2.3, 3.9]
During the past month, how much of the time did you have difficulty doing activities involving concentration and thinking?^h^	17.0	12.4	11.2 [8.6, 14.5]	5.8 [2.5, 8.5]
How much of the time, during the past month, did you have difficulty reasoning and solving problems; for example, making plans, making decisions, learning new things?^h^	10.8	7.4	6.2 [4.4, 8.8]	4.5 [2.0, 6.4]
**Summary variables (at least one limitation)**^**k**^				
Physical Functioning	30.1^11^	25.4	22.1 [18.6, 25.9]	8.0 [4.2, 11.5]
Role-Physical	33.9	27.9	25.7 [21.6, 30.2]	8.2 [3.7, 12.3]
Bodily Pain	18.4	14.7	13.5 [10.6, 17.1]	4.9 [1.3, 7.8]
Vitality	22.6	15.7	15.3 [12.1, 19.1]	7.3 [3.5, 10.5]
Social Functioning	14.5	10.9	11.3 [8.5, 14.9]	3.2 [-0.4, 6.0]
Role-Emotional	28.1	24.3	25.5 [21.3, 30.2]	2.6 [-2.2, 6.8]
Emotional Well-Being	16.5	12.5	11.1 [8.5, 14.4]	5.4 [2.1, 8.1]
Brain fog	19.8	15.3	13.8 [10.8, 17.4]	6.0 [2.4, 9.0]

^a^Fair/poor

^b^Somewhat worse/much worse than one year ago

^c^Yes, limited a lot

^d^Yes

^e^Quite a bit/Extremely

^f^Severe/very severe

^g^A little of the time/none of the time

^h^All of the time/most of the time

^i^Mostly false/definitely false

^j^Mostly true/definitely true

^k^Interpretation: 30.1% of cases report at least one limitation in physical functioning

^l^*N*=1374. Controls_STD_: Prevalence in controls standardized on cases characteristics. Δ_STD_=Cases- Controls_STD_

Table 4 presents the results of both bivariate and multivariable analyses comparing cases versus controls. Linear regression was used to analyze the PCS, MCS, and MHI scores, while logistic regression was employed to assess Self-Perceived Health Change. Cases had, on average and after adjustment for possible confounders, significantly lower PCS (β_PCS_=-2.8 [-3.7,-1.8]), MCS (β_MCS_=-2.4 [-3.4,-1.3]), and MHI (β_MHI_=-1.7 [-2.7,-0.7]) scores, compared to controls, indicating better quality of life in controls. Cases diagnosed between 6 and 9 months ago had non-significantly worse HRQoL than cases diagnosed more than 9 months ago (Table S1). To illustrate the effect of COVID-19 on HRQoL, we compared HRQoL among individuals affected by other medical conditions. Table S2 indicates that differences in HRQoL between cases and controls are comparable to those observed when comparing individuals with and without asthma. However, these differences are notably smaller than those observed when comparing individuals with and without chronic diseases (e.g., arthritis). (β_PCS_=-9.0 [-11.1,-6.8]). Multivariable analysis also showed HRQoL was similar in mild cases and controls, but was significantly lower for moderately ill and hospitalized cases. Table S3 presents additional analysis in participants recruited as controls, but who had COVID-19. Among this group, some individuals were found in the CCSS list (“diagnosed participants”), or were identified via antibodies testing (“undiagnosed cases”). Diagnosed participants had lower PCS, MCS and MHI scores and worse Self-Perceived Health Change compared to controls. Undiagnosed cases (controls who had COVID-19 antibodies) had similar results to controls who did not get COVID-19, showing a pattern comparable to mild cases.

**Table 4:** Bivariate and multivariable analysis of Health-Related Quality of Life Scores (PCS, MCS), Health Progression and Concentration Score (MIH) in controls and cases, overall and by severity

	**SF-36**			**MHI**^**c**^
	**PCS**^**a**^	**MCS**^**b**^	**Health Progression**	**Concentration score**
**Bivariate analysis**^**d**^	**Mean [95%CI]**	**Mean [95%CI]**	**%**	**Mean [95%CI]**
Controls	51.0 [50.4, 51.6]	51.0 [50.4, 51.6]	8.8%	50.7 [50.1, 51.3]
Cases (overall)	48.6 [47.8, 49.4]	48.5 [47.7, 49.3]	20.1%	49.0 [48.2, 49.8]
Cases: Mild	51.1 [50.1, 52.0]	50.6 [49.6, 51.6]	15.4%	50.1 [49.1, 51.1]
Cases: Moderate	45.2 [43.7, 46.7]	45.3 [43.8, 46.8]	27.7%	47.5 [46.0, 48.9]
Cases: Hospitalized	44.0 [39.8, 48.2]	46.6 [42.6, 50.6]	21.1%	46.9 [43.4, 50.3]
**Multivariable análisis**	β **[95%CI]**^**d**^	β **[95%CI]**^**d**^	**OR [95%CI]**^**e**^	β **[95%CI]**^**d**^
**Model 1**				
Controls: ref	0	0	1	0
Cases	-2.8 [-3.7, -1.8]***	-2.4 [-3.4, -1.3]***	3.2 [2.2, 4.7]***	-1.7 [-2.7, -0.7]***
**Model 2**				
Controls: ref	0	0	1	0
Mild	-0.6 [-1.7, 0.5]	-0.4 [-1.6, 0.8]	2.5 [1.6, 3.9]***	-0.6 [-1.8, 0.7]
Moderate	-5.8 [-7.2, -4.5]***	-5.2 [-6.6, -3.8]***	4.7 [2.9, 7.4]***	-3.1 [-4.6, -1.6]***
Hospitalized	-5.6 [-8.5, -2.7]***	-4.6 [-7.6, -1.6]***	2.1 [0.7, 7.0]	-4.2 [-7.3, -1.1]***

^a^Physical Component Summary (PCS)

^b^Mental Component Summary (MCS)

^c^Mental Health Inventory (MHI)

^d^Linear regressions models were employed to analyze the PCS, MCS and MHI scores.

^e^Logistic regression model was used for the Health Progression variable. Models were adjusted for comorbidities, decennial age, sex (male/female), province, education level (primary education or less / lower than secondary education / upper secondary education / higher education), working status (employee public or private / independent or informal workers / not working), current smoking status (current smoker, non-smoker), case-control status, and other sickness episode than COVID-19 during follow-up

^***^<0.01

^*^<0.05

Table 5 displays the multivariable analysis of HRQoL stratified by sex. Disparities between cases and controls were more pronounced in women than in men for the PCS (β=-3.9 [-5.3,-2.5] and -1.2 [-2.5,0.1] respectively), the MCS (β=-3.9 [-5.4,-2.5] and -0.3 [-1.7,1.0]), and the MHI scores (β=-2.3 [-3.8,-0.8] and -0.8 [-2.3-0.6]), but not in Self-Perceived Health Change. While scores were similar between controls and mild cases, they were lower in moderate/hospitalized cases compared to controls for both genders. However, the disparity between moderate/hospitalized cases and controls was greater among women compared to men.

**Table 5:** Multivariable analysis of Health-Related Quality of Life Scores (PCS, MCS), Health Progression and Concentration Score (MIH) in controls and cases, overall and by severity, stratified by sex

	**SF-36**			**MHI**^**c**^
	**PCS**^**a**^	**MCS**^**b**^	**Health Progression**	**Concentration**
	β **[95%IC]**^**d**^	β **[95%IC]**^**d**^	**OR [95%IC]**^**e**^	β **[95%IC]**^**d**^
**MEN**				
** Model 1**				
Controls: ref	0	0	1	0
Cases	-1.2 [-2.5, 0.1]	-0.3 [-1.7, 1.0]	2.9 [1.6, 5.3]***	-0.8 [-2.3, 0.6]
** Model 2**				
Controls: ref	0	0	1	0
Mild	0.6 [-1.0, 2.1]	1.5 [-0.1, 3.1]	1.9 [0.9, 4.1]	0.7 [-1.0, 2.4]
Moderate	-3.4 [-5.2, -1.5]***	-2.5 [-4.4, -0.6]***	5.5 [2.5, 12.0]***	-2.9 [-4.9, -0.8]***
Hospitalized	-3.6 [-6.8, -0.5]*	-3.4 [-6.7, -0.1]*	2.0 [0.5, 8.8]	-2.7 [-6.2, 0.9]
**WOMEN**				
** Model 1**				
Controls: ref	0	0	1	0
Cases	-3.9 [-5.3, -2.5]***	-3.9 [-5.4, -2.5]***	3.4 [2.1, 5.6]***	-2.3 [-3.8, -0.8]***
** Model 2**				
Controls: ref	0	0	1	0
Mild	-1.3 [-2.9, 0.3]	-1.8 [-3.5, -0.1]*	2.9 [1.6, 5.1]***	-1.5 [-3.2, 0.2]
Moderate	-7.7 [-9.6, -5.8]***	-7.2 [-9.2, -5.2]***	4.4 [2.4, 8.1]***	-3.0 [-5.0, -0.9]***
Hospitalized	-9.7 [-15.1, -4.2]***	-6.4 [-12.2, -0.7]*	1.7 [0.2, 19.0]	-7.5 [-13.4, -1.6]*

^a^Physical Component Summary (PCS)

^b^Mental Component Summary (MCS)

^c^Mental Health Inventory (MHI)

^d^Linear regressions models were employed to analyze the PCS, MCS and MHI scores

^e^Logistic regression model was used for the Health Progression variable. Models were adjusted for comorbidities, decennial age, sex (male/female), province, education level (primary education or less / lower than secondary education / upper secondary education / higher education), working status (employee public or private / independent or informal workers / not working), current smoking status (current smoker, non-smoker), case-control status, and other sickness episode than COVID-19 during follow-up

^***^<0.01

^*^<0.05

## Discussion

We observed decreased Health-Related Quality of Life and concentration scores in cases diagnosed with COVID-19 before vaccination, compared to those without COVID-19, 6 months to 2 years post-diagnosis. Cases reported 8.0% more physical limitations, 7.3% increased lack of vitality, and 6.0% more brain fog compared to controls with similar characteristics. 12.4% more cases than controls reported worse health compared to one year ago, indicating the percentage of unvaccinated cases whose COVID-19 infection significantly impacted HRQoL. Individuals with undiagnosed COVID-19 or mild symptoms had HRQoL similar to controls.

PCR-diagnosed cases exhibited lower HRQoL scores in the physical component summary, the mental health component summary (from the SF-36), and the concentration scale (from the Mental Health Inventory). These differences were statistically significant and persisted after adjusting for potential confounders. These findings align with existing literature on post-COVID conditions [34]. The findings indicating that recovered cases reported more physical limitations, fatigue, feeling worn out, and brain fog compared to controls are consistent with previous research [8, 35]. These self-perceptions confirm the substantial impact of COVID-19, with a higher proportion of cases reporting worse health compared with the year prior.

The difference in HRQoL between individuals who had COVID-19 and those who did not was primarily driven by women. This gender disparity, already observed, may be attributed to hormonal, immune differences, or reporting bias [36]. Individuals with moderate symptoms or who were hospitalized had lower HRQoL. Conversely, those with mild symptoms reported similar HRQoL to controls, as did undiagnosed cases identified by the presence of anti-SARS-CoV-2 antibodies. The comparison of controls by severity reveals that the more severe the acute illness, including those requiring hospitalization, the more significant the long-term effects are on all dimensions of HRQoL, particularly among women. This finding is consistent with existing literature, which suggests that the severity of the acute phase is linked to post-COVID conditions [6]. However, other factors may partially explain the difference between mild, moderate, and hospitalized cases in our study. Indeed, hospitalized cases exhibited differences compared to controls with respect to sex, age and comorbidities (Table S4). Moderate and hospitalized cases may have had poorer health before infection compared to those with mild symptoms, but our findings remained consistent after adjusting for reported comorbidities associated with COVID-19 severity. Hospitalization itself can directly affect patients, leading to general weakness and psychological effects [37, 38]. Both the severity of the acute phase and HRQoL were measured using self-reported questionnaires, and their association may be influenced by unmeasured confounders.

Regarding the COVID-19 symptoms, we found that from 4 months after diagnosis onwards, the percentage of cases who reported symptoms such as joint pain, chest tightness and skin manifestations stabilized at a higher level than in controls. The persistence of these symptoms has been reported in the literature [39, 40]. Other symptoms associated with the acute phase of COVID-19 and previously reported to be associated with PCC (fever, cough, headache, etc.) were not more prevalent. This might be explained by the longer time since diagnosis in our study compared to previous studies on PCC [1, 18]. Indeed, Montoy et al., recently showed that symptoms declined over time in both cases and controls, and at 12 months of follow-up, the reported symptoms were not significantly different [18].

The primary limitation of our study is the absence of a pre-COVID-19 HRQoL measure, which could have provided insight into the baseline health status. This limitation raises concerns regarding the possibility that individuals with better health prior to infection may have experienced fewer symptoms and thus been less likely to be diagnosed. However, sensitivity analyses conducted on controls who contracted COVID-19 (diagnosed or undiagnosed), yielded results consistent with the main analysis. Additionally, a higher proportion of individuals who had COVID-19 reported poorer health compared to the previous year, confirming the hypothesis of long-term impacts. Models were adjusted for health status previous infection using comorbidities, and few differences were observed in comorbidity prevalence between cases and controls. Furthermore, cases did not report more non-COVID-19 sickness episodes during follow-up compared to controls. Finally, it is worth noting that these analyses only applied to unvaccinated persons.

This study exhibits numerous strengths. It is a population-based cohort study that prospectively gathered data on COVID-19 cases and a contemporaneous matched group of uninfected individuals. With a large sample and a follow-up period ranging from 6 months to 2 years post-COVID-19 diagnosis (median follow-up of 9 months), this study allows for a comprehensive longitudinal analysis of diverse COVID-19 health impacts. Cases were drawn from the social security list, providing a representative sample of individuals affected by COVID-19 within the population. The community controls were matched to cases in terms of age, sex, and residence. Diagnosis was confirmed via PCR, with antibody analyses against the nucleocapsid (N) protein, distinguishing natural infection from vaccine-induced immunity. This approach facilitates the observation of differences in HRQoL among undiagnosed cases. Health outcomes were evaluated using validated questionnaires (SF-36, MHI) to assess the impact of COVID-19 on physical and mental health, as well as daily activities. The study encompasses a wealth of information and variables, enabling control for potential confounders, including chronic pathologies such as those causing chronic pain, allowing for a comparison of HRQoL scores between these conditions and COVID-19. Given the pandemic's characteristics and the varying prevalence of morbidities in LMICs [41], post-COVID conditions may vary, potentially affecting estimations and health system burdens.

## Conclusion

Long-term COVID-19 effects were confirmed, impacting physical and mental health. Those diagnosed pre-vaccination showed lasting HRQoL reduction (6 months to 2 years post-diagnosis), with greater physical limitations, reduced vitality, and increased brain fog. Women and severe cases were notably affected. Mild or undiagnosed cases didn't suffer long-term consequences. These findings provide reassurance, particularly given that most COVID-19 cases in many populations are mild. Considering significant disparities across countries, continued monitoring of COVID-19 progression and impact in LMICs remains imperative.

### Supplementary Information

Supplementary Material 1.

## Data Availability

The data that support the findings of this study are available from the Costa Rican Social Security Fund (CCSS) but restrictions apply to the availability of these data, which were used under license for the current study, and so are not publicly available. Anonymous data are however available from the authors upon reasonable request and with permission the CCSS.
