# Supplementary material for: COVID-19 and long-term impact on symptoms and Health-Related Quality of Life in Costa Rica: the RESPIRA cohort study
Source: BMC Infect Dis. 2024 Jun 4;24:557. doi: 10.1186/s12879-024-09450-6 (PMC11151688; doi:10.1186/s12879-024-09450-6)
Supplement: Supplementary file 1 — Supplementary Material 1. [file 12879_2024_9450_MOESM1_ESM.docx]

**Supplementary materials**

List of comorbidities included as adjustment in the variable “other comorbidities” in the models:

1. High blood pressure (Hypertension)
2. Type II Diabetes Mellitus (Non-autoimmune)
3. Obesity
4. Any type of cancer
5. High cholesterol and/or triglycerides (Dyslipidemia)
6. Asthma
7. Chronic Obstructive Pulmonary Disease (COPD)
8. Other respiratory disease
9. Arrhythmia (Cardiac rhythm disturbance)
10. Stroke (Cerebrovascular accident)
11. Congenital heart problems (congenital heart disease)
12. HIV - AIDS
13. Transplants
14. Other immunodeficiency
15. Systemic lupus erythematosus
16. Rheumatoid arthritis
17. Hashimoto's disease (Thyroiditis)
18. Type 1 Diabetes Mellitus
19. Multiple sclerosis
20. Other autoimmune disease
21. Kidney disease
22. Liver disease
23. Any other disease

**Table S1. Multivariable analysis of Health-Related Quality of Life Scores (PCS, MCS), Health Progression and Concentration Score (MIH) in controls and in cases according to time since diagnosis. Sensitivity analysis (N=1583).**

|  | **SF-36** | | | **MHI^3^** |
| --- | --- | --- | --- | --- |
|  | **PCS^1^** | **MCS^2^** | **Health Progression** | **Concentration** |
| **Bivariate analysis^4^** | **Mean [95%IC]** | **Mean [95%IC]** | **%** | **Mean [95%IC]** |
| *Controls* | 51.0 [50.4, 51.6] | 51.0 [50.4, 51.6] | 8.8% | 50.7 [50.1, 51.3] |
| *Cases* |  |  |  |  |
| *Diagnosed between 6 and 9 months ago* | 48.4 [47.2, 49.5] | 48.0 [46.8, 49.2] | 21.7% | 49.0 [47.9, 50.0] |
| *Diagnosed more than 9 months ago* | 48.9 [47.6, 50.1] | 49.1 [47.9, 50.3] | 14.0% | 49.0 [47.8, 50.3] |
|  |  |  |  |  |
| **Multivariable analysis** | β **[95%IC]^4^** | β **[95%IC] ^4^** | **OR [95%IC] ^5^** | β **[95%IC] ^4^** |
| **Model 1** |  |  |  |  |
| *Controls: ref* | 0 | 0 | 1 | 0 |
| *Cases* |  |  |  |  |
| *Diagnosed between 6 and 9 months ago* | -3.2 [-4.4, -2.0]*** | -2.6 [-3.9, -1.4]*** | 3.6 [2.4, 5.4]*** | -1.5 [-2.7, -0.2]* |
| *Diagnosed more than 9 months ago* | -2.3 [-3.5, -1.1]*** | -2.0 [-3.3, -0.8]*** | 2.0 [1.0, 3.9] | -2.0 [-3.3, -0.6]*** |

*Legend. ^1^Physical Component Summary (PCS). ^2^Mental Component Summary (MCS). ^3^Mental Health Inventory (MHI). ^4^Linear regressions models were employed to analyze the PCS, MCS and MHI scores. ^5^Logistic regression model was used for the Health Progression variable. Models were adjusted for comorbidities, decennial age,* *sex (male/female), province, education level (primary education or less / lower than secondary education / upper secondary education / higher education), working status (employee public or private / independent or informal workers / not working), current smoking status (current smoker, non-smoker), case-control status, and other sickness episode than COVID-19 during follow-up. ***<0.01, *<0.05*

**Table S2: Benchmark to observe the impact of certain comorbidities on HRQoL**

|  | **PCS^1^** | **MCS^2^** | **Health Progression** | **MHI^3^** |
| --- | --- | --- | --- | --- |
|  | β **[95%IC]^4^** | β **[95%IC] ^4^** | **OR [95%IC]^5^** | β **[95%IC] ^4^** |
| **Other comorbidities** |  |  |  |  |
| Hypertension | -3.0 [-4.1,-1.8]*** | -1.8 [-3.0,-0.6]*** | 1.1 [0.7-1.7] | -0.8 [-2.1,0.4] |
| Diabetes II | -1.2 [-2.7,0.2] | -0.4 [-1.9,1.1] | 1.1 [0.6-1.8] | 0.6 [-1.0,2.2] |
| Obesity | -1.6 [-2.9,-0.2]* | -0.3 [-1.6,1.1] | 1.1 [0.7-1.8] | 0.7 [-0.8,2.1] |
| Cholesterol | -1.0 [-2.1,0.3] | -1.6 [-2.7,-0.4]*** | 1.0 [0.7-1.5] | -1.8 [-2.9,-0.6]*** |
| Asthma | -3.6 [-4.9,-2.3]*** | -1.2 [-5.5,-2.7]*** | 1.6 [1.0-2.5]* | -1.9 [-3.3,-0.4]* |
| Comorbidity including pain^6^ | -9.0 [-11.1,-6.8]*** | -5.4 [-7.6,-3.2]*** | 4.1 [2.2-7.5]*** | -2.1 [-4.4,0.2] |
| Comorbidity including impact on mental health^7^ | -2.7 [-5.5,0.2] | -5.9 [-8.9,-3.0]*** | 2.3 [0.9-5.4] | -1.9 [-5.0,1.2] |

*^1^Physical Component Summary (PCS). ^2^Mental Component Summary (MCS). ^3^Mental Health Inventory (MHI). ^4^Linear regressions models were employed to analyze the PCS, MCS and MHI scores. ^5^Logistic regression model was used for the Health Progression variable. Models were adjusted for comorbidities, decennial age,* *sex (male/female), province, education level (primary education or less / lower than secondary education / upper secondary education / higher education), working status (employee public or private / independent or informal workers / not working), current smoking status (current smoker, non-smoker), case-control status, and other sickness episode than COVID-19 during follow-up. ^6^Comorbidity including pain: hernia, osteoarthritis, low back pain, fibromyalgia, rheumatic fever, rheumatoid arthritis, facial paralysis (N=74). ^7^Comorbidity including impact on mental health: anxiety, depression, panic attack (N=40).***<0.01, *<0.05.*

**Table S3. Multivariable analysis of Health-Related Quality of Life in controls. Sensitivity analysis (N=1354).**

|  | **SF-36** | | | **MHI^3^** |
| --- | --- | --- | --- | --- |
|  | **PCS^1^** | **MCS^2^** | **Health Progression** | **Concentration** |
| **Bivariate analysis** | **Mean [95%IC]** | **Mean [95%IC]** | **%** | **Mean [95%IC]** |
| *Controls* | 51.0 [50.4, 51.6] | 51.0 [50.4, 51.6] | 8.8% | 50.7 [50.1, 51.3] |
| *Controls with COVID-19 (overall)* | 49.0 [47.9, 50.1] | 49.1 [48.0, 50.2] | 14.1% | 49.3 [48.3, 50.4] |
| *Diagnosed before recruitment* | 47.0 [44.9, 49.0] | 48.1 [46.2, 50.0] | 16.8% | 49.2 [47.2, 51.1] |
| *Diagnosed after recruitment* | 47.9 [45.9, 49.9] | 46.8 [44.7, 48.9] | 18.2% | 46.8 [44.9, 48.8] |
| *Undiagnosed (antibodies only)* | 51.7 [50.1, 53.3] | 52.1 [50.5, 53.6] | 8.1% | 51.7 [50.2, 53.2] |
|  |  |  |  |  |
| **Multivariable analysis** | β **[95%IC]^4^** | β **[95%IC]^4^** | **OR [95%IC]^5^** | β **[95%IC]^4^** |
| **Model 1** |  |  |  |  |
| *Controls: ref* | 0 | 0 | 1 | 0 |
| *Controls with COVID-19 (overall)* | -2.2 [-3.3, -1.1]*** | -1.8 [-2.9, -0.7]*** | 1.8 [1.2, 2.6]*** | -1.5 [-2.7, -0.4]* |
|  |  |  |  |  |
| **Model 2** |  |  |  |  |
| *Controls: ref* | 0 | 0 | 1 | 1 |
| *Diagnosed before recruitment* | -3.4 [-5.1, -1.7]*** | -2.4 [-4.1, -0.6]*** | 2.1 [1.2, 3.6]*** | -1.5 [-3.3, 0.3] |
| *Diagnosed after recruitment* | -3.2 [-4.9, -1.5]*** | -3.5 [-5.2, -1.8]*** | 2.3 [1.4, 4.0]*** | -3.6 [-5.4, -1.8]*** |
| *Undiagnosed (antibodies only)* | -0.3 [-1.9, 1.3] | 0.3 [-1.4, 1.9] | 1.1 [0.6, 2.1] | 0.4 [-1.3, 2.1] |

*Legend. ^1^Physical Component Summary (PCS). ^2^Mental Component Summary (MCS). ^3^Mental Health Inventory (MHI). ^4^Linear regressions models were employed to analyze the PCS, MCS and MHI scores. ^5^Logistic regression model was used for the Health Progression variable. Models were adjusted for comorbidities, decennial age,* *sex (male/female), province, education level (primary education or less / lower than secondary education / upper secondary education / higher education), working status (employee public or private / independent or informal workers / not working), current smoking status (current smoker, non-smoker), case-control status.*

**Table S4. Demographic, socioeconomic characteristics and comorbidities in cases at recruitment (overall and by severity)**

|  | **Cases (N=641)** | | | | |
| --- | --- | --- | --- | --- | --- |
|  | **Mild** | **Moderate** | **p (Mild vs Moderate)** | **Hospitalized** | **p (Mild vs Hospitalized)** |
|  | 379 (59%) | 223 (35%) |  | 39 (6%) |  |
| **Sex** |  |  | p_1_=NS |  | p_2_<0.01 |
| Men | 166 (44%) | 93 (42%) |  | 27 (69%) |  |
| Women | 213 (56%) | 130 (58%) |  | 12 (31%) |  |
| **Age** |  |  | p_1_=0.03 |  | p_2_<0.01 |
| 18-29y | 79 (21%) | 36 (16%) |  | 2 (5%) |  |
| 30-39y | 74 (20%) | 46 (21%) |  | 1 (3%) |  |
| 40-49y | 64 (17%) | 51 (23%) |  | 4 (10%) |  |
| 50-59y | 57 (15%) | 46 (21%) |  | 13 (33%) |  |
| 60-69y | 64 (17%) | 32 (14%) |  | 10 (26%) |  |
| 70y+ | 41 (11%) | 12 (5%) |  | 9 (23%) |  |
| **Time since diagnosis at recruitment** |  |  | p_1_=NS |  | p_2_=NS |
| 0-29 days | 138 (36%) | 73 (33%) |  | 8 (21%) |  |
| 30-89 days | 82 (22%) | 55 (25%) |  | 8 (21%) |  |
| 90 days or more | 159 (42%) | 95 (43%) |  | 23 (59%) |  |
| **Province** |  |  | p_1_=NS |  | p_2_=NS |
| GAM | 245 (65%) | 144 (65%) |  | 30 (77%) |  |
| Guanacaste-Puntarenas | 134 (35%) | 79 (35%) |  | 9 (23%) |  |
| **Education level** |  |  | p_1_=NS |  | p_2_=0.03 |
| Complete Elementary School | 102 (27%) | 63 (28%) |  | 19 (49%) |  |
| Incomplete High School | 68 (18%) | 45 (20%) |  | 3 (8%) |  |
| Complete High School or Technical training | 78 (21%) | 51 (23%) |  | 5 (13%) |  |
| Complete or Incomplete University | 131 (35%) | 64 (29%) |  | 12 (31%) |  |
| **Working status** |  |  | p_1_=NS |  | p_2_=NS |
| Employee (public or private) | 167 (44%) | 106 (48%) |  | 10 (26%) |  |
| Independent or informal workers | 54 (14%) | 36 (16%) |  | 8 (21%) |  |
| Not working | 158 (42%) | 81 (36%) |  | 21 (54%) |  |
| **Currently smoking*** | 27 (7%) | 14 (6%) | p_1_=NS | 1 (3%) | p_2_=NS |
| **Comorbidities** |  |  |  |  |  |
| Hypertension | 105 (28%) | 65 (29%) | p_1_=NS | 23 (59%) | p_2_<0.01 |
| Diabetes II | 49 (13%) | 25 (11%) | p_1_=NS | 13 (33%) | p_2_<0.01 |
| Obesity | 57 (15%) | 37 (17%) | p_1_=NS | 7 (18%) | p_2_=NS |
| Cholesterol | 99 (26%) | 69 (31%) | p_1_=NS | 15 (38%) | p_2_=NS |
| Asthma | 47 (12%) | 43 (19%) | p_1_=0.02 | 8 (21%) | p_2_=NS |
| Another comorbidity related to COVID-19 | 45 (12%) | 34 (15%) | p_1_=NS | 6 (15%) | p_2_=NS |
| Another comorbidity | 98 (26%) | 63 (28%) | p_1_=NS | 13 (33%) | p_2_=NS |
| **Other sickness episode than COVID-19 during follow-up**** | 115 (30%) | 88 (39%) | p_1_=0.02 | 17 (44%) | p_2_=NS |

**Table S5. Scoring coefficients Uued for SF-36 Mental and Physical Scales (N=1588)**

|  | **Mental** | **Physical** |
| --- | --- | --- |
| Physical Functioning | 0.01 | 0.25 |
| Role-Physical | 0.04 | 0.25 |
| Bodily Pain | 0.02 | 0.27 |
| General Health | 0.08 | 0.18 |
| Vitality | 0.20 | 0.17 |
| Social Functioning | 0.23 | 0.04 |
| Role-Emotional | 0.18 | 0.03 |
| Emotional Well-Being | 0.39 | -0.01 |

Reference: Farivar SS, Cunningham WE, Hays RD. Correlated physical and mental health summary scores for the SF-36 and SF-12 Health Survey, V.1. Health Qual Life Outcomes. 2007;5(1):54.
